# Draft Genome Sequence of *Virgibacillus* sp. Strain AGTR, Isolated from Hypersaline Lake Acıgöl in Turkey

**DOI:** 10.1128/mra.00555-22

**Published:** 2022-08-31

**Authors:** Meryem Menekse Kılıc, Nurgul Balci, Nevin Gul Karaguler

**Affiliations:** a Istanbul Technical University, Faculty of Science and Letters, Department of Molecular Biology and Genetics, Istanbul, Turkey; b Istanbul Topkapi University, Plato Vocational School, Medical Laboratory Techniques Program, Istanbul, Turkey; c Istanbul Technical University, Dr. Orhan Öcalgiray Molecular Biology, Biotechnology, and Genetics Research Center, Istanbul, Turkey; d Istanbul Technical University, Department of Geological Engineering, Geomicrobiology and Biogeochemistry Laboratory, Istanbul, Turkey; Montana State University

## Abstract

*Virgibacillus* sp. strain AGTR, which is a haloalkaliphilic microorganism, was isolated from a sediment sample collected in hypersaline Lake Acıgöl in Turkey. It has the potential to produce biotechnologically essential proteases. Here, the whole-genome sequence and its annotations are reported.

## ANNOUNCEMENT

The use of enzymes from halophiles in industrial applications is not limited to their stability at high salt concentrations, as these extremozymes are also tolerant to high temperatures or low temperatures and alkali pH and are stable in the presence of organic solvents (1–4). *Virgibacillus* sp. strain AGTR is a Gram-positive, endospore-forming, motile, and halophilic microorganism that was isolated from hypersaline Lake Acıgöl (the salinity rate varies between 5.8% and 13% [wt/vol]). Sediment samples were collected from hypersaline Lake Acıgöl in Turkey (37.837100N, 29.861E). Sediment samples were diluted, and 100 μL diluted sample was plated on protease activity screening plates, which are nutrient agar (NA) plates containing 1% (wt/vol) skim milk and 10% NaCl (wt/vol). The plates were incubated at 30°C for 7 days. Colonies were isolated by repeated streaking (three times) on fresh screening plates. The isolate showing the maximum hydrolysis zone was selected for whole-genome sequencing. The isolate was cultivated in 100 mL nutrient broth (NB) (with 10% NaCl) at 30°C for 24 h, and DNA isolation was carried out with a MoBio kit (catalog number 12888-50). One hundred nanograms of genomic DNA was used to create sequencing libraries with the TruSeq Nano DNA low-throughput library preparation kit (catalog number 20015964; Illumina). Quality control, in terms of size distribution and quantity of the libraries, was performed by using a 2100 Bioanalyzer (Agilent Technologies, USA). Sequencing by synthesis (SBS) was performed using the HiSeq Rapid SBS kit v2 (catalog number FC-402-4023; Illumina). The Illumina HiSeq 2500 platform was used for sequencing. The raw sequence data were checked with FastQC (5), in terms of sequence quality and the presence of any adapter sequences. A total of 4,625,053 raw reads were obtained.

Raw paired-end sequencing data were used for *de novo* assembly with the Unicycler genome assembler v0.4.8 (6). The assembled genome size was 4,708,499 bp, with a GC content of 36.66%, which is in good accordance with values for previously published *Virgibacillus* genomes. The assembled genome was annotated using Prokka v1.13 (7). The annotation pipeline with Prokka uses several external tools, such as Prodigal (8) for coding sequences, RNAmmer v1.2 (9) for rRNA genes, ARAGORN (10) for tRNA genes, and Infernal v1.1 (11) for noncoding RNAs. The draft genome was predicted to have 4,536 coding sequences, 64 tRNA genes, and 5 rRNA sequences. Assembled genome features are indicated in Table 1. The average nucleotide identity (ANI) was calculated by using EzBioCloud TruBac ID software v1 (12), which revealed 99.44% similarity to Virgibacillus marismortui. Default parameters were used for all software unless otherwise noted.

**TABLE 1:** Genome features of *Virgibacillus* sp. strain AGTR

Feature	Finding
Total no. of contigs	215
Total length (bp)	4,708,499
Length of largest contig (bp)	209,032
*N*_50_ (bp)	50,902
*N*_75_ (bp)	27,280
*L*_50_	27
*L*_75_	58
No. of coding genes	4,536
Mean sequencing coverage (×)	291.70

### Data availability.

The whole-genome sequence of *Virgibacillus* sp. strain AGTR was submitted to GenBank under BioProject accession number PRJNA701885, BioSample accession number SAMN17916045, and nucleotide accession number JAJERH000000000. The raw data were deposited in the Sequence Read Archive (SRA) under SRA accession number SRP376786.
